# AMPK-Activated Protein Kinase Suppresses Ccr2 Expression by Inhibiting the NF-κB Pathway in RAW264.7 Macrophages

**DOI:** 10.1371/journal.pone.0147279

**Published:** 2016-01-22

**Authors:** Fumiaki Kumase, Kimio Takeuchi, Yuki Morizane, Jun Suzuki, Hidetaka Matsumoto, Keiko Kataoka, Ahmad Al-Moujahed, Daniel E. Maidana, Joan W. Miller, Demetrios G. Vavvas

**Affiliations:** 1 Retina Service, Angiogenesis Laboratory, Massachusetts Eye and Ear Infirmary, Department of Ophthalmology, Harvard Medical School, Boston, Massachusetts, United States of America; 2 Department of Ophthalmology, Okayama University Graduate School of Medicine, Dentistry and Pharmaceutical Sciences, Okayama, Japan; 3 Department of Ophthalmology, Tokyo Medical University, Tokyo, Japan; University of Patras, GREECE

## Abstract

C-C chemokine receptor 2 (Ccr2) is a key pro-inflammatory marker of classic (M1) macrophage activation. Although Ccr2 is known to be expressed both constitutively and inductively, the full regulatory mechanism of its expression remains unclear. AMP-activated protein kinase (AMPK) is not only a master regulator of energy homeostasis but also a central regulator of inflammation. In this study, we sought to assess AMPK’s role in regulating RAW264.7 macrophage Ccr2 protein levels in resting (M0) or LPS-induced M1 states. In both M0 and M1 RAW264.7 macrophages, knockdown of the AMPKα1 subunit by siRNA led to increased Ccr2 levels whereas pharmacologic (A769662) activation of AMPK, attenuated LPS-induced increases in Ccr2 expression in an AMPK dependent fashion. The increases in Ccr2 levels by AMPK downregulation were partially reversed by NF-κB inhibition whereas TNF-a inhibition had minimal effects. Our results indicate that AMPK is a negative regulator of Ccr2 expression in RAW264.7 macrophages, and that the mechanism of action of AMPK inhibition of Ccr2 is mediated, in part, through the NF-κB pathway.

## Introduction

Macrophages play a key role in the innate immune response and help to direct the acquired immune response. The acute phase of inflammation is associated with pro-inflammatory classical (M1) macrophage activation. The resolution phase of inflammation is associated with alternatively activated (M2) macrophages, which exhibit an anti-inflammatory phenotype [1]. M1 macrophages are activated by treatment with IFNγ or LPS, and M2 macrophages are activated by treatment with Th2 cytokines IL-4 or IL-13. Switch to the M2 phenotype can be enhanced by IL-10. Macrophages can also be skewed during differentiation in vitro, and the resultant phenotype depends upon the cytokine provided to support their differentiation [2]. Recently, different regulatory pathways have been shown to be associated with either the M1 or M2 activation states. They involve a variety of molecular machineries at the genomic, transcriptomic, and post-transcriptomic levels [3].

C-C chemokine receptor 2 (Ccr2) is the primary receptor for monocyte chemoattractant protein 1/chemokine ligand 2 (MCP1/CCL2), a member of chemokine family of proteins. Ccr2 is expressed on monocytes and macrophages, where it serves as a crucial recruitment factor by directing cells to sites of injury and inflammation [4]. Ccr2 is one of the M1 macrophage phenotype markers [5–8] and has been shown to be involved in macrophage-dependent inflammatory responses in various chronic inflammatory diseases, including atherosclerosis, Alzheimer disease, uveitis, and choroidal neovascularization [9–12] Ccr2 is expressed at the cell surface both variably and under stringent regulation [13]; however, the underlying mechanisms are obscure.

AMP-activated protein kinase (AMPK) is a serine/threonine kinase that regulates energy homeostasis and metabolic stress [14]. AMPK acts as a sensor of cellular energy status and maintains the balance between ATP production and consumption. In mammals, AMPK exists as a heterotrimer with α, β, and γ subunits, each of which is encoded by two or three genes (α1, α2, β1, β2, γ1, γ2, and γ3). The α subunit possesses catalytic activity, whereas the β and γ subunits are regulatory and maintain the stability of the heterotrimer complex. Phosphorylation of α subunit at Thr^172^ is essential for AMPK activation [15]. In macrophages, AMPKα1 is the predominant isoform expressed, whereas AMPKα2 expression is negligible [16].

Previous work by others [17] and our group [18–22] suggests that AMPK has a much wider range of functions. Over the past several years, a role of AMPK in the regulation of inflammatory response has been revealed [23]. Because AMPK’s functions are closely linked to macrophage polarization skewing [16,24], we hypothesized that AMPK regulates Ccr2 expression in macrophages. To address this in the present study, we utilized the macrophage cell line RAW264.7 and investigated the role of AMPKα1 in regulating Ccr2 expression in the LPS-treated (M1) or untreated (M0) state.

## Materials and Methods

### Antibodies and reagents

All antibodies for Western blotting were purchased from Cell Signaling (Beverly, MA) except AMPKα1, β-actin, and TATA (Abcam, Cambridge, MA) and AMPKα2 (Santa Cruz biotechnology, Santa Cruz, CA). A pharmacological AMPK activator (A769662) and the inhibitor of NF-κB (LY303511) were purchased from Tocris Bioscience (Ellisville, MO). The inhibitors for IKK (BMS345541) and NF-κB (SM7368) were purchased from Sigma-Aldrich. LPS (ultrapure LPS, *E*. *coli* 0111: B4) was purchased from InvivoGen (San Diego, CA, USA). Recombinant mouse TNF-α protein, anti-mouse TNF-α neutralizing antibody and isotype control IgG were obtained from R&D Systems (Abingdon, UK).

### Cell cultures

The mouse macrophage cell line RAW264.7 was obtained from American Type Culture Collection (ATCC, Manassas, VA) and cultured in Dulbecco’s modified Eagle’s medium (DMEM) (ATCC) containing 2% heat-inactivated fetal bovine serum (FBS) and 1% penicillin/streptomycin (Invitrogen, Carlsbad, CA). RAW264.7 macrophages were either treated (M1) or untreated (M0) with LPS for 12 h. For all experiments, cells were grown at 37°C in a humidified atmosphere of 5% CO_2_ and 95% air.

### Flow cytometry

Single-cell suspensions of RAW264.7 macrophages were stained with PE- conjugated anti-Ccr2 (R&D Systems, Abingdon, UK). PE-IgG2B (R&D Systems) was used as the matched isotype control. Stained cells were analyzed with a flow cytometer (LSR II; Becton-Dickinson, Franklin Lakes, NJ) and a commercial program (Summit v4.3; Dako Colorado, Inc., Fort Collins, CO). Normalized median fluorescence intensities (MFI) were calculated by dividing median fluorescence intensity of Ccr2 by that of isotype control.

### Protein extraction, subcellular fraction, and Western blotting

Protein extraction, subcellular fraction, Western blotting, and densitometry were carried out as described previously [22].

### Small interfering RNA (siRNA) transfection

RAW264.7 macrophages were transfected with ON-TARGETplus Mouse Prkaa1 siRNA (a mixture of four Prkaa1 siRNAs) (Dharmacon/Thermo Scientific, Lafayette, CO) or ON-TARGETplus Non-targeting Pool (a mixture of four negative control siRNAs) (Dharmacon/Thermo Scientific) utilizing HiPerFect transfection reagent according to manufacture’s recommended protocols (Qiagen, Valencia, CA). The medium was changed 24 h after transfection. The transfection efficiency was determined by Western blot and densitometric analysis of bands 4 days after siRNA transfections.

### Statistical analysis

All experiments were repeated a minimum of three times. All data were expressed as means ± S.E. Depending on the experiment, a Student’s, one-way ANOVA, or two-way ANOVA test was performed, and Bonferroni post hoc correction was applied for multiple comparisons using GraphPad Prism soft ware (GraphPad, La Jolla, CA). Differences were considered significant at *p <* 0.05.

## Results

### AMPKα1 regulates Ccr2 expression in RAW264.7 macrophages

**Fig 1:**
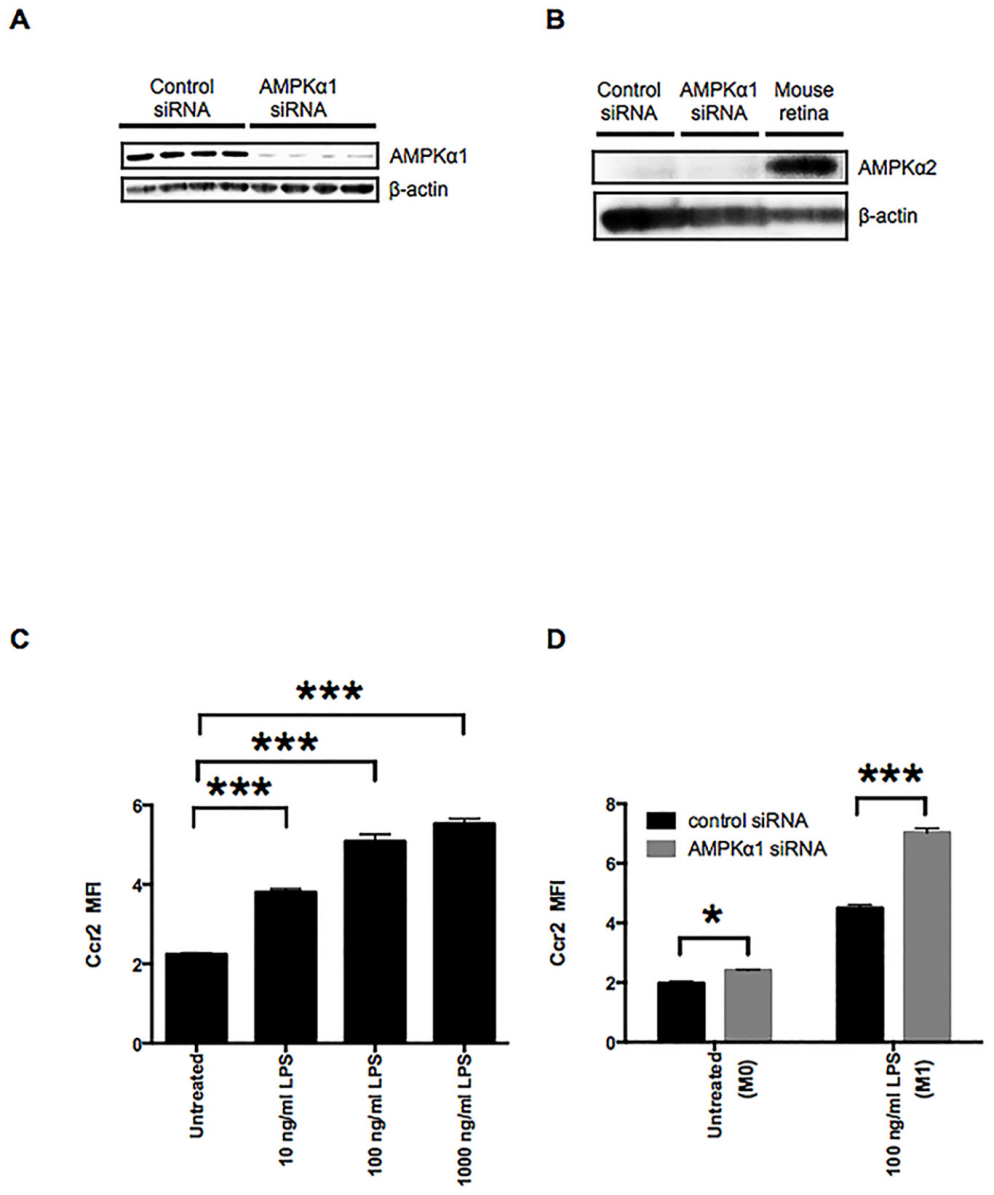
AMPKα1 negatively regulates Ccr2 expression in the M0 and the LPS-stimulated M1 macrophages. A: Reduced AMPKα1 protein levels in macrophages treated with AMPKα1 siRNA RAW264.7 were confirmed by Western blotting of whole cell lysates. β-actin was probed as an internal control. B: Whole cell lysates of RAW264.7 macrophages treated with either control or AMPKα1 siRNA were examined by Western blotting to confirm compensation by AMPKα2. Tissue lysates prepared from mouse retina were used as a positive control for expression of AMPKα2 protein. β-actin was probed as an internal control. C: Ccr2 expression on RAW264.7 macrophages was analyzed by flow cytometry. RAW264.7 macrophages were stimulated with 10–1000 ng/ml of LPS for 12 h. D: Flow cytometry analysis of Ccr2 expression on RAW264.7 macrophages treated with either control or AMPKα1 siRNA. RAW264.7 macrophages were stimulated with 100 ng/ml of LPS for 12 h to induce the M1 state. n = 3. ***, *p <* 0.001.

To study the function of AMPK in regulating macrophage Ccr2 expression, we used the macrophage cell line RAW264.7 and downregulated the catalytically active AMPKα1 subunit with siRNA. Knockdown of AMPKα1 suppressed AMPKα1 protein levels by approximately 90% (Fig 1A) without detectable compensation by AMPKα2 (Fig 1B). Knockdown of AMPKα1 was associated with decreased total and total phospho-AMPKα (S1 Fig), indicating that AMPKα1 is the predominant α isoform in RAW264.7 macrophages. LPS induction of the M1 state increases Ccr2 expression in a dose-dependent manner (Fig 1C). Knockdown of AMPK using siRNA was associated with increased LPS-induced Ccr2 expression in both LPS-treated (M1) and untreated (M0) RAW264.7 macrophages (Fig 1D). These results indicate that AMPKα1 is inhibitory for Ccr2 expression.

### Pharmacological activation of AMPK inhibits Ccr2 expression in RAW264.7 macrophages in the M1 state

**Fig 2:**
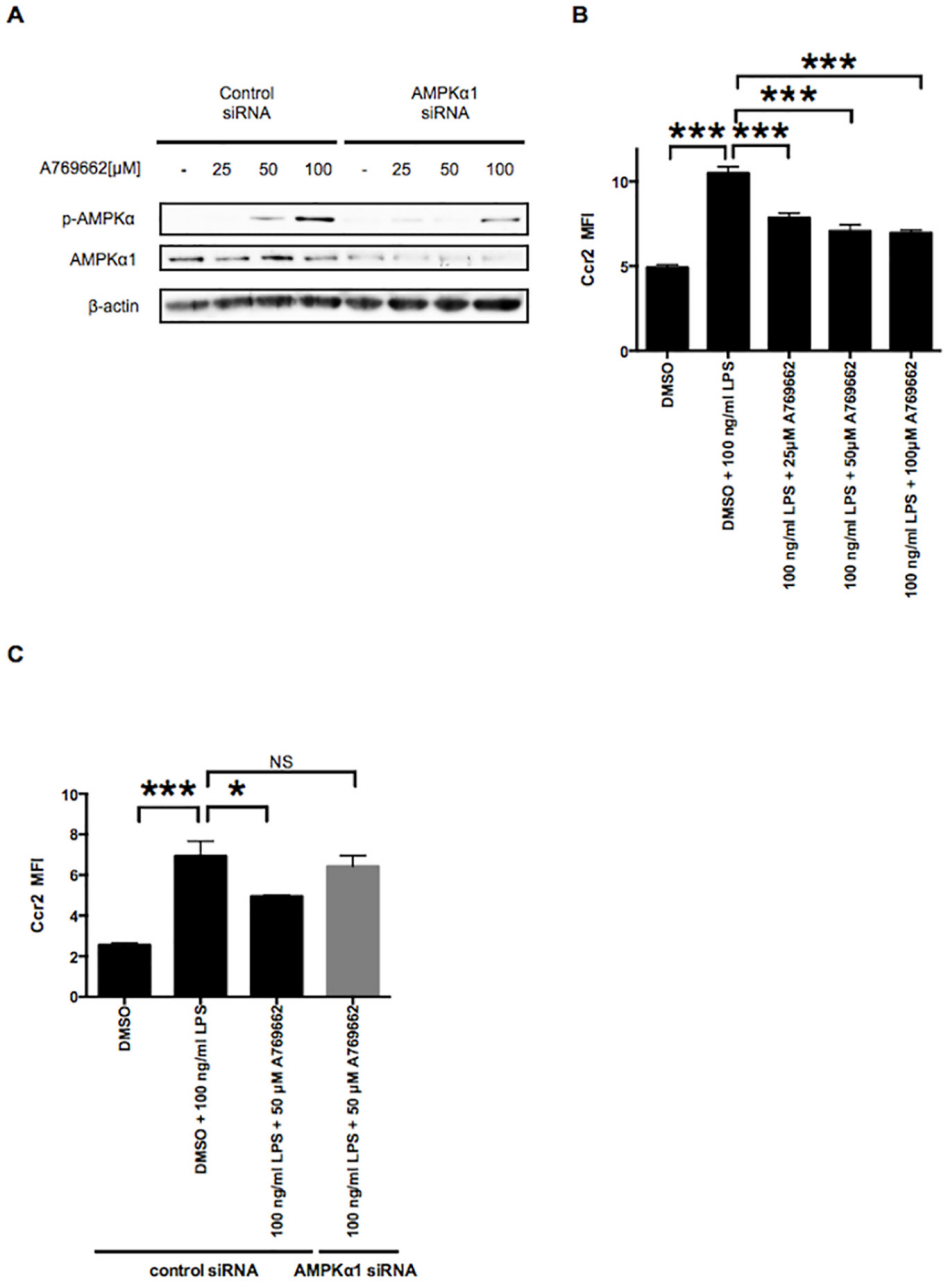
Pharmacological activation of AMPK counter-regulates Ccr2 expression in the LPS-stimulated M1 macrophages. A: RAW264.7 macrophages treated with either control or AMPKα1 siRNA were additionally treated with 25–100 μM of the AMPK activator, A769662. The phosphorylation of AMPKα (p-AMPKα) after A769662 treatment was examined by Western blotting. β-actin was probed as an internal control. B: RAW264.7 macrophages were pretreated with 25–100 μM A769662 for 2 h, followed by co-treatment with 100 ng/ml of LPS and each different concentration of A769662 for 12 h. Dimethyl sulfoxide (DMSO) was used as a control. Ccr2 expression was analyzed by flow cytometry. C: RAW264.7 macrophages treated with either control or AMPKα1 siRNA were pretreated with 50 μM A769662 for 2 h, followed by co-treatment with 100 ng/ml of LPS and 50 μM A769662 for 12 h. DMSO was used as a control. Ccr2 expression was analyzed by flow cytometry. n = 3. *, *p <* 0.05; ***, *p <* 0.001.

Although 5-amino-4-imidazole carboxamide riboside (AICAR) is used extensively as an AMPK activator, the effects of AICAR have been shown to be mostly independent of AMPK in macrophages [25]. Therefore, we tested the more specific AMPK activator, A769662 [26,27]. A769662 treatments led to increased phosphorylation of AMPKα in a dose-dependent manner in control siRNA-treated RAW264.7 macrophages. In contrast, AMPKα phosphorylation was attenuated in RAW264.7 macrophages treated with AMPKα1 siRNA (Fig 2A). Application of A769662 inhibited Ccr2 expression in a dose-dependent manner in the LPS-induced M1 state of RAW264.7, further indicating that activation of AMPKα1 inhibits RAW264.7 Ccr2 expression (Fig 2B). This inhibitory effect of A769662 was lost if cells were pretreated with AMPKα1 siRNA but not with control scrambled siRNA (Fig 2C). These results suggest that A769662 inhibits Ccr2 expression in an AMPK-dependent manner in the M1 state of RAW264.7 macrophages.

### Loss of AMPKα1 leads to increased expression of Ccr2 in RAW264.7 macrophages through the NF-κB pathway in the M0 state

**Fig 3:**
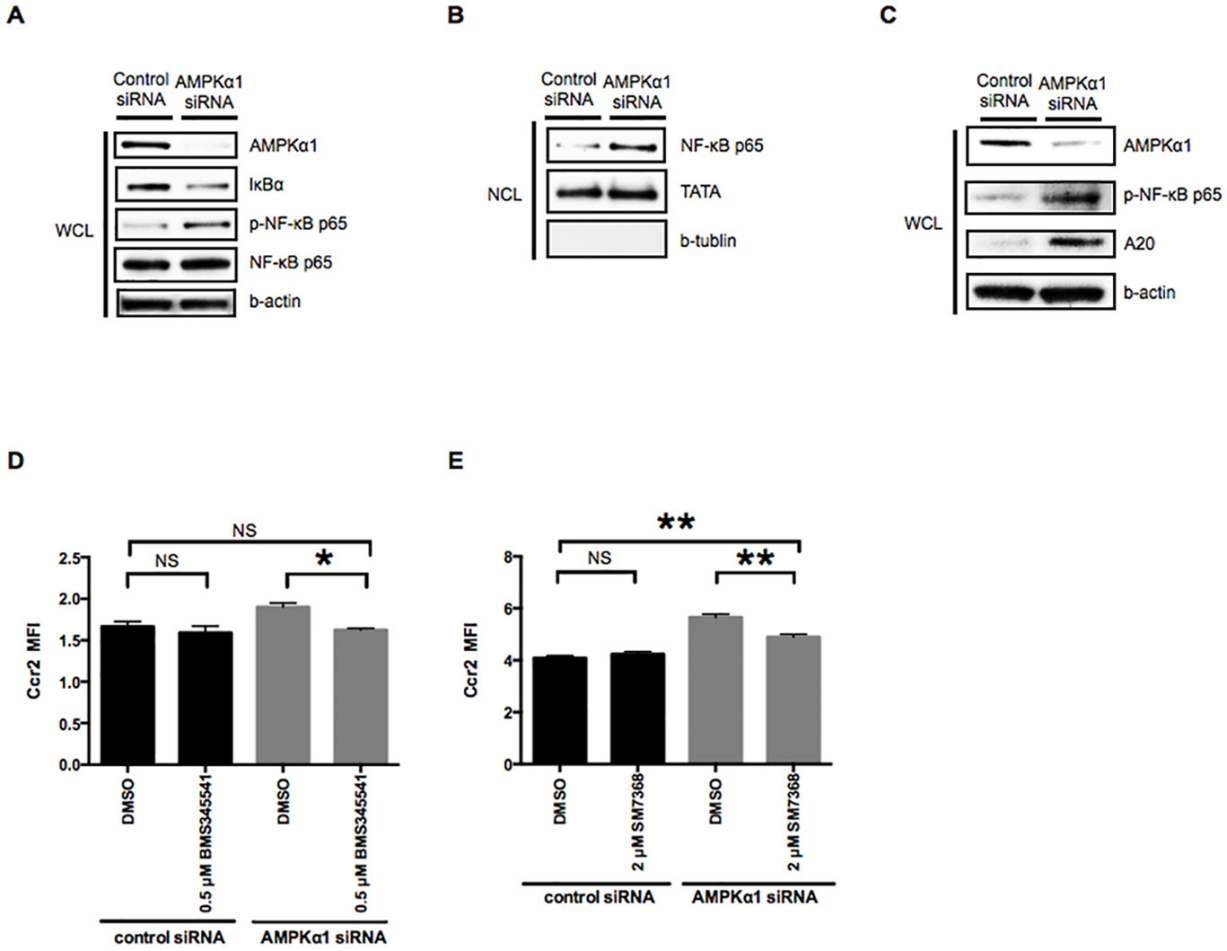
Activation of the NF-κB pathway reverses AMPKα1-dependent downregulation of Ccr2 expression in M0 macrophages. A: Whole cell lysates (*WCL*) of RAW264.7 macrophages treated with either control or AMPKα1 siRNA were examined by Western blotting to determine IκBα degradation and NF-κB p65 phosphorylation. β-actin was probed as an internal control. B: Nuclear cell lysates (*NCL*) of RAW264.7 macrophages treated with either control or AMPKα1 siRNA were examined by Western blotting to determine activation of a NF-κB pathway. TATA and β-tubulin antibodies were used to confirm equal protein loading and to assess the relative purity of the nuclear cell lysates. C: Whole cell lysates (*WCL*) of RAW264.7 macrophages treated with either control or AMPKα1 siRNA were examined by Western blotting to determine the expression of A20. β-actin was probed as an internal control. D and E: RAW264.7 macrophages treated with either control or AMPKα1 siRNA were additionally treated with IKK inhibitor (BMS345541, 0.5 μM) and NF-κB inhibitor (SM7368, 2 μM) for 12 h. Ccr2 expression was analyzed by flow cytometry. n = 3. *, *p <* 0.05; **, *p <* 0.01.

Previous studies have suggested that the NF-κB pathway is negatively regulated by AMPK [23]. To investigate whether the NF-κB pathway is involved in the AMPK-dependent downregulation of Ccr2 expression, we used Western blotting to examine the effects of AMPKα1 reduction on the NF-κB pathway. IκBα was degraded and the phosphorylation of NF-κB p65 was increased in RAW264.7 macrophages treated with AMPKα1 siRNA (Fig 3A). Nuclear extracts from RAW264.7 macrophages treated with AMPKα1 siRNA (but not control siRNA) showed an increase in the nuclear translocation of NF-κB p65 (Fig 3B). We next investigated the expression of A20 (or cylindromatosis), a deubiquinase with NF-κB-dependent transcription [28]. The expression of A20 was markedly higher in RAW264.7 macrophages treated with AMPKα1 siRNA compared to control-treated RAW264.7 macrophages (Fig 3C). These results suggest that AMPKα1 reduction causes activation of the NF-κB pathway.

To determine whether activation of the NF-κB pathway is responsible for the upregulation of Ccr2 expression in RAW264.7 macrophages treated with AMPKα1 siRNA, we used an IκBα kinase inhibitor (BMS345541) and an inhibitor of NF-κB activation (SM7368) and evaluated Ccr2 expression by flow cytometry. Treatments with either BMS345541 or SM7368 significantly suppressed the upregulation of Ccr2 expression associated with AMPKα1 knockdown (Fig 3D and 3E). These results indicate that activation of NF-κB pathway reverses AMPKα1-dependent downregulation of Ccr2 expression in M0 RAW264.7 macrophages.

### AMPKα1 reduction amplifies increased Ccr2 expression in RAW264.7 macrophages through the NF-κB pathway in the M1 state

**Fig 4:**
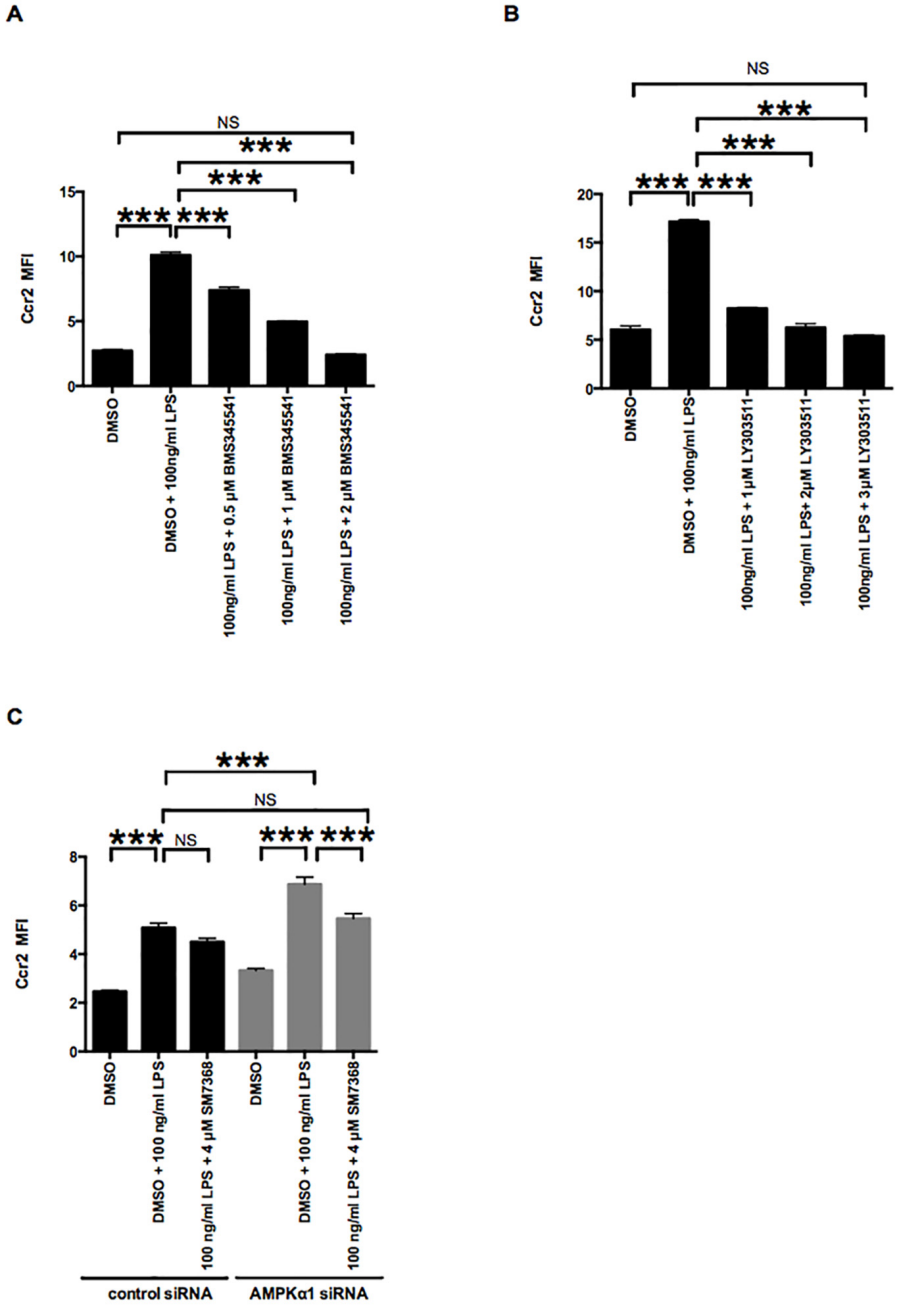
AMPKα1 reduction increases Ccr2 expression in the LPS-stimulated M1 macrophages through NF-κB signaling. A, B, C and D: RAW264.7 macrophages were pretreated with IKK inhibitor (BMS345541, 0.5–2 μM) and NF-κB inhibitors (LY303511, 1–3 μM; SM7368, 4 μM) for 2 h, followed by co-treatment with 100 ng/ml of LPS and different concentration of each inhibitor for 12 h. Ccr2 expression was analyzed by flow cytometry. n = 3. ***, *p <* 0.001.

Our results indicated that AMPKα1 reduction leads to the increase of Ccr2 expression by activating NF-κB pathway, which prompted us to examine whether the NF-κB pathway is responsible for upregulating Ccr2. To this end, we inhibited the NF- κB pathway in RAW264.7 macrophages after treatment with control or AMPKα1 siRNA in the LPS-induced M1 state. Treatment with BMS345541 (inhibitor of I kappa B kinase [29]) or LY303511 (inhibitors of NF-κB activation [30,31]) did not affect macrophage viability (S2 Fig) but significantly suppressed increased Ccr2 expression in the M1 state (Fig 4A and 4B). These results suggest that Ccr2 expression is likely mediated via the NF-κB pathway in the M1 state. Treatment with SM7368 didn’t alter LPS-induced Ccr2 expression in RAW264.7 macrophages treated with control siRNA. In contrast, treatment with SM7368 significantly inhibited the increase of LPS-induced Ccr2 expression associated with AMPKα1 siRNA treatment. These results suggest that AMPKα1 amplifies LPS-induced Ccr2 expression through the NF-κB pathway in the M1 state.

### Autocrine or paracrine TNF-α is minimally involved in increased Ccr2 expression in M1 RAW264.7 macrophages induced by AMPKα1 downregulation

Since AMPK deletion leads to NF-κB activation and NF-κB activation can lead to increased TNF-α levels [32–34] and deletion of AMPK can further upregulate TNF-induced effects [22]we wanted to investigate if the increased Ccr2 expression by AMPKα1 down-regulation is mediated through TNF-α. For this reason we used an inhibitory antibody to TNF-α to block the autocrine or paracrine effects of TNF-α from RAW264.7 macrophages after treatment with control or AMPKα1 siRNA in the M0 state and the LPS-induced M1 state. Treatment with anti-TNF-α neutralizing antibody minimally suppressed increased Ccr2 expression by AMPKα1 down-regulation in the M1 state but not in M0 state (S3 Fig). TNF-α administration alone lead to a mild dose-dependent increase of Ccr2 expression in RAW264.7 macrophages (S4 Fig).These results indicate that increased Ccr2 expression by AMPKα1 down-regulation is independent of TNF-α in the M0 state, and only partially dependent on TNF-α in the M1 state.

## Discussion

In the present study, we have shown that AMPKα1 regulates Ccr2 expression in RAW264.7 macrophages in both the M0 and the M1 states. Furthermore, we showed that AMPKα1 downregulation amplifies Ccr2 expression in RAW264.7 macrophages through the NF-κB signaling pathway. In our study, Ccr2 expression is increased in LPS-stimulated M1 RAW264.7 macrophages (Fig 1C) similarly to LPS-stimulated neutrophils [35], but in contrast to other studies suggesting downregulation of Ccr2 expression in monocyte/macrophages by LPS via mechanisms involving receptor internalization and degradation as well as a reduction in *Ccr2* mRNA stability [36–40]. We speculate that these differences might be due to the different time points (up to 4 h in the previous studies vs. 12 h in our present study) and/or cell type [30].

Our results indicate for the first time that AMPKα1 is a novel negative regulator of Ccr2 expression via NF-κB pathway in RAW264.7 macrophages. We demonstrated that the pharmacological AMPK activator, A769662, suppresses Ccr2 expression in RAW264.7 macrophages in an AMPK-dependent fashion, and that AMPKα1 reduction by siRNA leads increased Ccr2 expression in RAW264.7 macrophages in the M0 and LPS-stimulated M1 states (Figs 1D and 2C). Sag *et al*. [16] reported that dominant-negative inactivation of AMPKα1 induces TNF-α and IL-6 in the LPS-stimulated M1 state, whereas constitutive activation of AMPKα1 inhibits production of these proinflammatory cytokines in M1 macrophages; this was the first implication of AMPKα1 as a potent regulator of functional macrophage polarization. Similarly, Yang *et al*. [41] reported that inactivation of AMPKα1 by short hairpin RNA or dominant-negative AMPKα1 increases TNF-α mRNA in the M0 and the LPS-stimulated M1 state, suggesting that AMPKα1 is a key determinant of basal inflammatory signaling and an important suppressor of LPS-induced inflammation in macrophages. We demonstrated that using anti-TNF-α neutralizing antibody, TNF-α is minimally involved in Ccr2 upregulation in RAW264.7 macrophages by AMPKα1 down-regulation (S3 Fig). Mounier *et al*. [24] reported that genetic deletion of AMPKα1 attenuates expression of CD206, a phenotypic M2 marker, in the IL-4 stimulated M2 state, suggesting AMPKα1 is required for acquisition of an M2 phenotype in macrophages. Thus, although additional studies are needed, our work (together with these prior studies) implicates AMPKα1 as an important regulator of functional and phenotypic polarization of macrophages.

Our data are consistent with the notion that Ccr2 is associated with M1 macrophages [42] and that NF-κB is important for the expression of M1-specific cytokines [1,43,44]. In our study, we showed that AMPKα1 suppresses Ccr2 expression in RAW264.7 macrophages by inhibiting the NF-κB pathway in the M0 and M1 state. Furthermore, the NF-κB inhibitor SM7368 reversed the effects of AMPKα1 knockdown on Ccr2 (Figs 3E and 4C). This study provides new evidence that AMPKα1 plays a role in modulating Ccr2 expression in RAW264.7 macrophages, at least partly, through the NF-κB pathway. This AMPKα1 reduction (and resultant NF-κB activation) in RAW264.7 M0 macrophages is in line with our earlier work, in which we showed that deletion of AMPKα results in constitutive NF-κB activation in mouse embryonic fibroblasts (MEFs) [22]. In the current investigation, we found that AMPKα1 reduction in RAW 264.7 macrophages increases the degradation of IκBα, leading to increased phosphorylation of NF-κB p65, its nuclear translocation, and the expression of A20 (an NF-κB-induced negative feedback regulator) [28] (Fig 3A, 3B and 3C). Thus, our current work (together with our earlier work) suggests that AMPKα plays a key role in restricting NF-κB to a cytoplasmic location in resting M0 macrophages.

Many studies have suggested that the activation of AMPK inhibits the NF-κB signaling pathway through multiple mechanisms [16,41,45–48], although in most cases these were not in cells of the immune system [23]. AMPKα1 has been shown to suppress NF-κB signaling indirectly via its downstream mediator SIRT1 in macrophages [41]. Although the mechanism by which AMPKα1 regulates NF-κB is not fully elucidated, there is a possibility that AMPK inhibits IKK-dependent IκBα phosphorylation either directly or indirectly. Phosphorylation of IκBα by the upstream kinase IKK is essential for NF-κB nuclear translocation [49]. Our results demonstrated that AMPKα1 reduction leads to the degradation of IκBα and nuclear translocation of NF-κB, indicating that AMPKα1 either directly targets IKK activity, or targets another factor upstream of IKK. Previous studies have demonstrated a role of AMPK as an inhibitor of IKKβ activity [22,48,50–52]. Further study is required to determine the target of AMPKα1 for interference with the NF-κB pathway.

A769662 is a small-molecule, direct AMPK activator that does not increase cellular AMP or ADP, but acts instead by directly binding AMPK at activation site(s) [53]. Recently, salicylates (which are among the oldest medical compounds known to humankind) have been found to activate AMPK by direct binding to the same site as A769662 [54]. Some direct AMPK activators have been slated for human clinical trials for type 2 diabetes [55], and it remains unclear whether the beneficial effects of long-term salicylate treatment on insulin resistance are mediated by inhibiting of NF-κB signaling, by AMPK activation, or by a combination of these pathways [56]. Galic *et al*. showed a causal role for macrophage AMPK in the development of insulin resistance [57], and Weisberg *et al*. showed the importance of Ccr2 in the development of insulin resistance [4]. We showed that A769662 attenuates LPS-induced Ccr2 expression (Fig 2B), which is likely mediated via the NF-κB pathway (Fig 4A and 4B) in an AMPK-dependent manner in RAW264.7 macrophages (Fig 2C). Although additional studies are needed, our findings (together with the previous works of Galic *et al*. and Weisberg *et al*.) raise a possibility that the beneficial effects of direct AMPK activators or salicylate-based drugs on inflammation and insulin resistance may be mediated, at least in part, by modulating macrophage CCR2 expression via NF-κB signaling.

**Fig 5:**
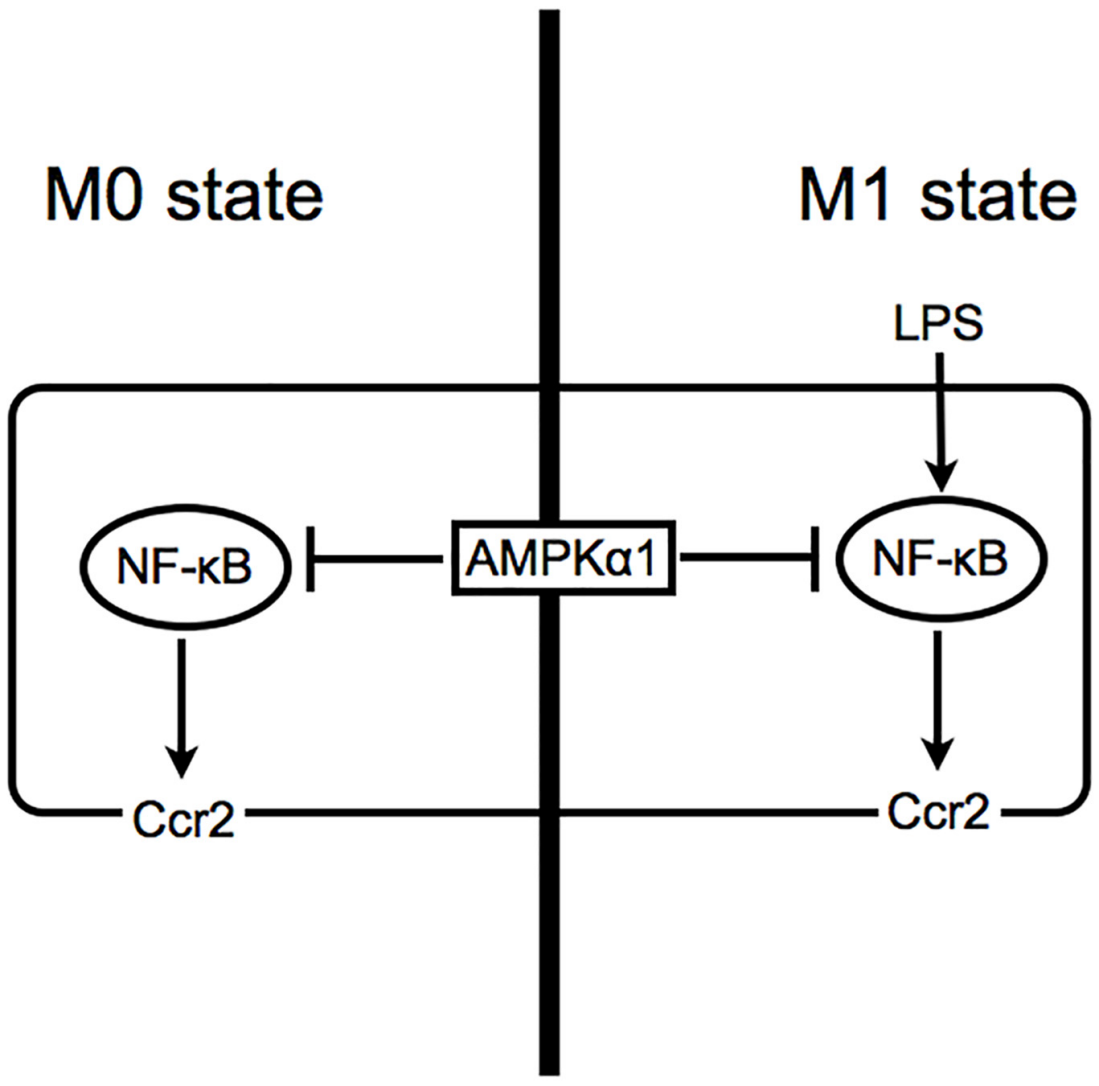
Proposed mechanism of regulation for AMPKα1 in Ccr2 expression in RAW264.7 macrophages. AMPKα1 negatively regulates Ccr2 expression by inhibiting NF-κB pathway in both M0 and M1 macrophages.

In conclusion, we identified AMPKα1 as a novel negative regulator of Ccr2 expression in RAW264.7 macrophages at least in part through the NF-κB pathway (Fig 5). Because Ccr2 and AMPK each plays an important role in numerous inflammatory diseases, our findings might provide fundamental insights not only into the regulatory mechanism of Ccr2 expression and the function of AMPK, but also into the pathogenesis of inflammatory diseases.

## Supporting Information

S1 Fig AMPKα1 reduction leads to decreased total and phospho-AMPKα. Whole cell lysates of RAW264.7 macrophages treated with either control or AMPKα1 siRNA were examined by Western blotting to examine the levels of AMPKα (p-AMPKα), AMPKα1, and total AMPKα. β-actin was probed as an internal control.
(TIFF)

S2 Fig Effects of NF-κB inhibitors on the cell viability of RAW264.7 macrophages in the absence or presence of LPS. A and C: RAW264.7 macrophages were treated with NF-κB inhibitors (SM7368, 1–4μM; LY303511, 1–3 μM) for 12 h. The cell viability was assessed by MTT assay. n = 3. *, *p <* 0.05. B and D: RAW264.7 macrophages were pretreated with NF-κB inhibitors (SM7368, 1–4μM; LY303511, 1–3 μM) for 2 h, followed by co-treatment with 100 ng/ml of LPS and different concentration of each inhibitor for 12 h. The cell viability was assessed by MTT assay. n = 3. *, *p <* 0.05; **, *p <* 0.01.
(TIFF)

S3 Fig Increased Ccr2 expression by AMPKα1 down-regulation is independent of TNF-α in the M0 state and only partially dependent on TNF-α in the M1 state. RAW264.7 macrophages treated with either control or AMPKα1 siRNA were co-treated with 10 ng/ml of control isotype IgG or 10 ng/ml of TNF-α neutralizing antibody in the absence or presence of 100 ng/ml of LPS for 12 h. Ccr2 expression was analyzed by flow cytometry. n = 3. **, *p <* 0.01; ***, *p <* 0.001.
(TIFF)

S4 Fig TNF-α administration alone leads to a mild dose-dependent increase of Ccr2 expression in. RAW264.7 macrophages. Ccr2 expression on RAW264.7 macrophages was analyzed by flow cytometry. RAW264.7 macrophages were stimulated with 1–50 ng/ml of TNF-α for 12 h. Ccr2 expression was analyzed by flow cytometry. n = 3. **, *p <* 0.01.
(TIFF)
