# Correction: AMPK-Activated Protein Kinase Suppresses Ccr2 Expression by Inhibiting the NF-κB Pathway in RAW264.7 Macrophages

**DOI:** 10.1371/journal.pone.0304894

**Published:** 2024-05-30

**Authors:** Fumiaki Kumase, Kimio Takeuchi, Yuki Morizane, Jun Suzuki, Hidetaka Matsumoto, Keiko Kataoka, Ahmad Al-Moujahed, Daniel E. Maidana, Joan W. Miller, Demetrios G. Vavvas

In Fig 2, the beta actin is incorrect. Please see the correct Fig 2 here.

**Fig 2:**
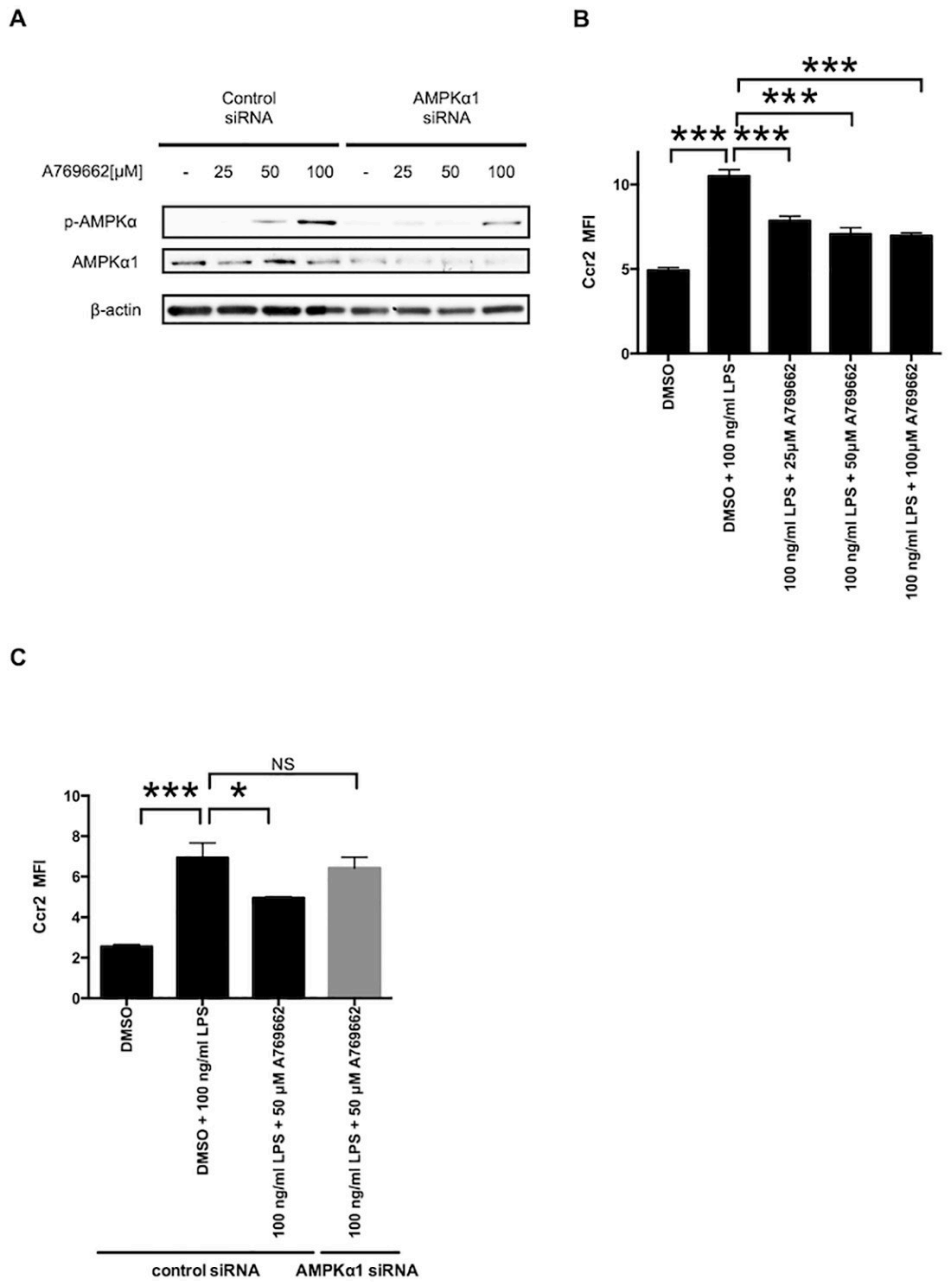
Pharmacological activation of AMPK counter-regulates Ccr2 expression in the LPS-stimulated M1 macrophages. A: RAW264.7 macrophages treated with either control or AMPKα1 siRNA were additionally treated with 25–100 μM of the AMPK activator, A769662. The phosphorylation of AMPKα (p-AMPKα) after A769662 treatment was examined by Western blotting. β-actin was probed as an internal control. B: RAW264.7 macrophages were pretreated with 25–100 μM A769662 for 2 h, followed by co-treatment with 100 ng/ml of LPS and each different concentration of A769662 for 12 h. Dimethyl sulfoxide (DMSO) was used as a control. Ccr2 expression was analyzed by flow cytometry. C: RAW264.7 macrophages treated with either control or AMPKα1 siRNA were pretreated with 50 μM A769662 for 2 h, followed by co-treatment with 100 ng/ml of LPS and 50 μM A769662 for 12 h. DMSO was used as a control. Ccr2 expression was analyzed by flow cytometry. n = 3. *, p < 0.05; ***, p < 0.001.

## References

[1] Kumase F, Takeuchi K, Morizane Y, Suzuki J, Matsumoto H, Kataoka K, et al. (2016) AMPK-Activated Protein Kinase Suppresses Ccr2 Expression by Inhibiting the NF-κB Pathway in RAW264.7 Macrophages. PLoS ONE 11(1): e0147279. doi: 10.1371/journal.pone.0147279 26799633 PMC4723067

