# Supplementary figures and images for: AMPK-Activated Protein Kinase Suppresses Ccr2 Expression by Inhibiting the NF-κB Pathway in RAW264.7 Macrophages
Source: PLoS One. 2016 Jan 22;11(1):e0147279. doi: 10.1371/journal.pone.0147279 (PMC4723067; doi:10.1371/journal.pone.0147279)

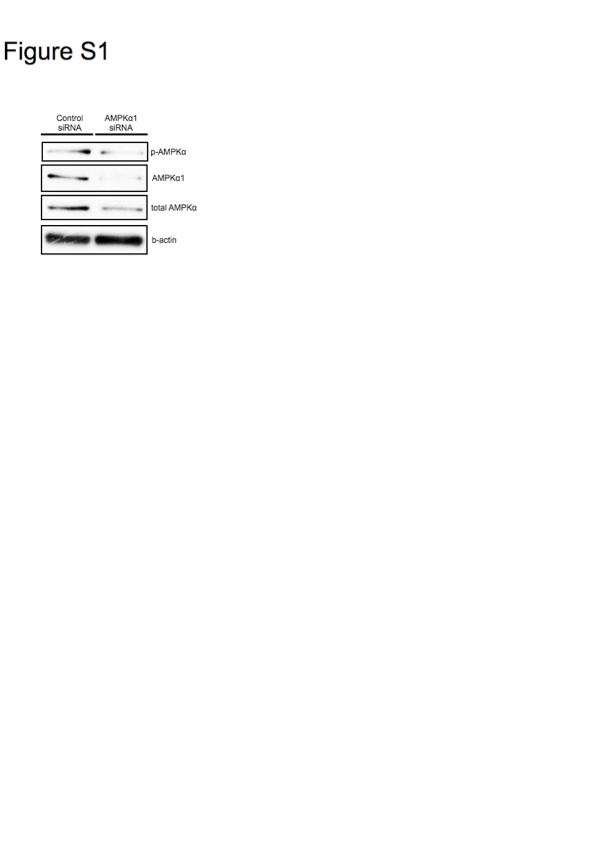

Supplement: S1 Fig — Whole cell lysates of RAW264.7 macrophages treated with either control or AMPKα1 siRNA were examined by Western blotting to examine the levels of AMPKα (p-AMPKα), AMPKα1, and total AMPKα. β-actin was probed as an internal control. (TIFF) [file pone.0147279.s001.tiff]

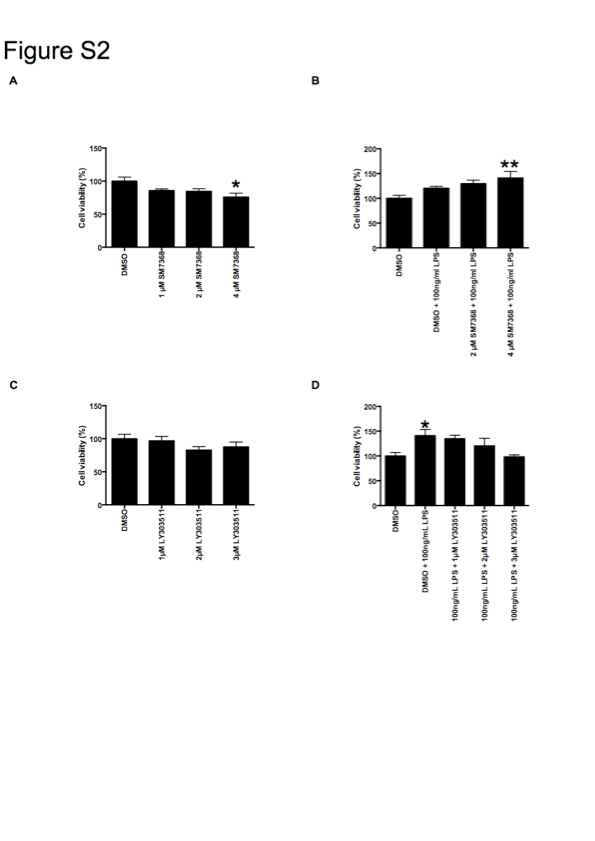

Supplement: S2 Fig — A and C: RAW264.7 macrophages were treated with NF-κB inhibitors (SM7368, 1–4μM; LY303511, 1–3 μM) for 12 h. The cell viability was assessed by MTT assay. n = 3. *, p < 0.05. B and D: RAW264.7 macrophages were pretreated with NF-κB inhibitors (SM7368, 1–4μM; LY303511, 1–3 μM) for 2 h, followed by co-treatment with 100 ng/ml of LPS and different concentration of each inhibitor for 12 h. The cell viability was assessed by MTT assay. n = 3. *, p < 0.05; **, p < 0.01. (TIFF) [file pone.0147279.s002.tiff]

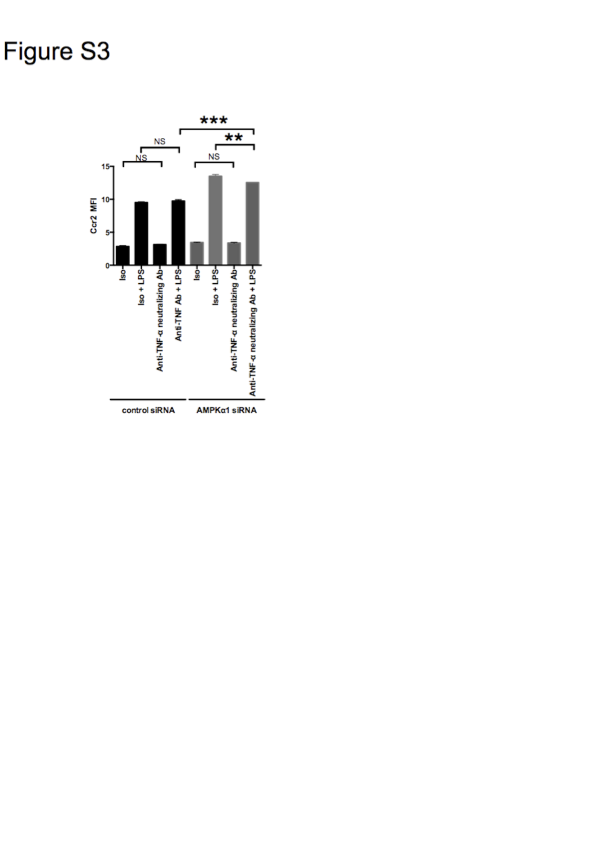

Supplement: S3 Fig — RAW264.7 macrophages treated with either control or AMPKα1 siRNA were co-treated with 10 ng/ml of control isotype IgG or 10 ng/ml of TNF-α neutralizing antibody in the absence or presence of 100 ng/ml of LPS for 12 h. Ccr2 expression was analyzed by flow cytometry. n = 3. **, p < 0.01; ***, p < 0.001. (TIFF) [file pone.0147279.s003.tiff]

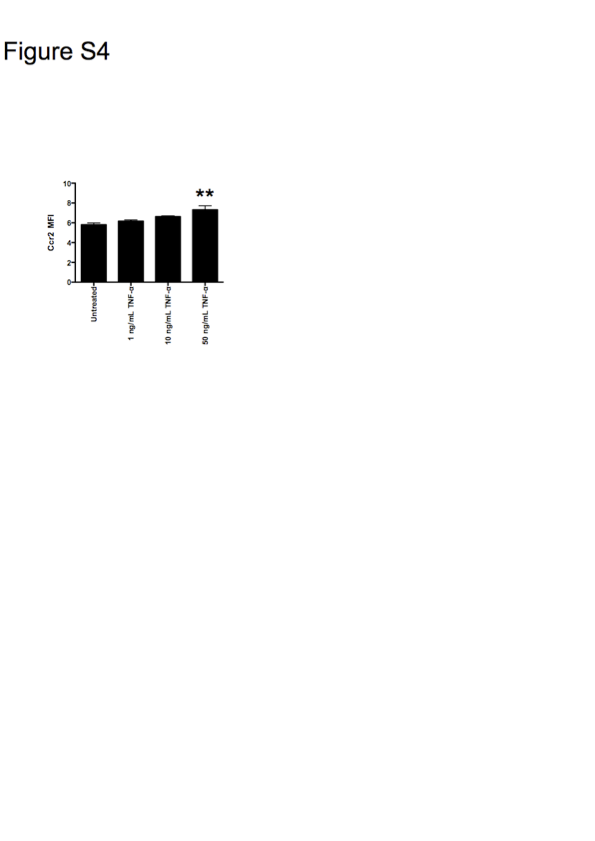

Supplement: S4 Fig — Ccr2 expression on RAW264.7 macrophages was analyzed by flow cytometry. RAW264.7 macrophages were stimulated with 1–50 ng/ml of TNF-α for 12 h. Ccr2 expression was analyzed by flow cytometry. n = 3. **, p < 0.01. (TIFF) [file pone.0147279.s004.tiff]
